# Evaluation of tongue/mandible volume ratio in children with obstructive sleep apnea

**DOI:** 10.1590/2177-6709.23.4.072-078.oar

**Published:** 2018

**Authors:** Kavita Hotwani, Krishna Sharma, Arpan Jaiswal

**Affiliations:** 1 VSPM Dental College and Research Center, Department of Pediatric and Preventive Dentistry (Nagpur/MH, India). VSPM Dental College and Research Center Department of Pediatric and Preventive Dentistry NagpurMH India; 2 Toothart Comprehensive Dental Care (Nagpur/MH, India). Toothart Comprehensive Dental Care NagpurMH India; 3 Sharad Pawar Dental College (Wardha/MH, India). Sharad Pawar Dental College WardhaMH India

**Keywords:** Tongue/mandible ratio, MRI, Volumetric, Children

## Abstract

**Objective:**

The present study was an attempt to investigate tongue/mandible volume ratio in children, using volumetric magnetic resonance imaging (MRI) for early screening and to aid in treatment planning.

**Methods:**

Volumetric evaluation of tongue volume/mandible volume ratio (TV/MV ratio) in children with obstructive sleep apnea (OSA) using MRI was carried out retrospectively on available DICOM MR images of children in the age group of 10-14 years. MRI image records of patients diagnosed with OSA were obtained from interventional radiology department records, at Sharad Pawar Dental College and Hospital (Datta Meghe Institute of Medical Sciences, Nagpur/India). The age, gender, height and weight of the subjects were retrieved from patient database and registered. For the control group, available MRI images of healthy subjects without OSA were retrieved. Body mass index (BMI) was also calculated using the height and the weight present in the records. Measurements from MR images were made using DICOM image processing software. Soft tissue and bony structure segmentation was performed by manual tracing. The tongue volume and mandible volume were directly computed using the software. The tongue volume/mandible volume ratio (TV/MV) was generated using the above values and expressed as a percentage for both groups.

**Results:**

The difference between OSA group and control group with respect to TV/MV ratio was found to be highly significant at 0.05 level of significance. There was no significant correlation between BMI and TV/MV ratio in OSA group (*p*= 0.451) as well as in control group (*p*= 0.094).

**Conclusion:**

TV/MV ratio may be an appropriate variable to evaluate the risk of OSA, representing the balance between skeletal morphology and soft tissue morphology in craniofacial complex.

## Resumo

**Objetivo:**

o presente estudo foi uma tentativa de investigar a relação volumétrica língua/mandíbula em crianças, usando imagens de ressonância magnética (IRM), para triagem inicial e como meio auxiliar no plano de tratamento.

**Métodos:**

a avaliação da relação volume da língua/volume da mandíbula (relação VL/VM) em crianças com apneia obstrutiva do sono (AOS) utilizando IRM foi realizada retrospectivamente em imagens de RM em formato DICOM disponíveis de crianças na faixa etária de 10 a 14 anos. As documentações com IRM de pacientes com diagnóstico de AOS foram obtidas nos arquivos do departamento de radiologia da *Sharad Pawar Dental College and Hospital (Datta Meghe Institute of Medical Sciences,* Nagpur/Índia*)*. A idade, o sexo, altura e peso dos indivíduos foram obtidos a partir dos registros dos pacientes. Para o grupo controle, foram selecionadas imagens de RM disponíveis de indivíduos saudáveis sem AOS. O índice de massa corporal (IMC) também foi calculado a partir da altura e do peso constantes nos registros. As medições nas imagens de RM foram feitas usando um *software* de processamento de imagens DICOM. A segmentação entre tecidos moles e estrutura óssea foi realizada por delineamento manual. O volume da língua e o volume da mandíbula foram calculados diretamente com o *software*. A relação volume da língua/volume da mandíbula (VL/VM) foi calculada usando os valores acima e expressa em porcentagens, para ambos os grupos.

**Resultados:**

a diferença entre o grupo com AOS e o grupo controle, quanto à relação VL/VM, foi altamente significativa ao nível de significância de 0,05. Não houve, porém, correlação significativa entre o IMC e a relação VL/VM, nem no grupo AOS (*p*= 0,451), nem no grupo controle (*p*= 0,094).

**Conclusão:**

a relação VL/VM pode ser uma variável apropriada para se avaliar o risco de AOS, representando o equilíbrio entre a morfologia esquelética e a morfologia dos tecidos moles no complexo craniofacial.

## INTRODUCTION

Obstructive sleep apnea syndrome (OSAS) is a congregation of conditions in which breathing stops intermittently and repeatedly for ten or more seconds during sleep.^1^ OSAS is anticipated to be associated with increased risk of hypertension, cardiovascular disease, stroke, daytime sleepiness, motor vehicle accidents, and diminished quality of life. It is considered that the causal site of the disorders is in the upper airway, which is theoretically divided into the nasal cavity; the area including the adenoids, soft palate and palatine tonsils; and the posterior part of the tongue. 

OSAS with its origin in the posterior part of the tongue occurs when the tongue muscles relax during sleep and the tongue falls backward to obstruct the airway.^1^ The following neuromuscular response to altered breathing have been reported in literature:^2^

1. Altered tongue function and posture.
2. Altered mandibular position and dimension.

Solow and Kreiborg^3^ stated in their “soft tissue-stretching hypothesis” that the postural relationship of head and tongue is altered after birth to maintain the airway. A link between respiratory mode and development of malocclusion could be due to this altered soft tissue stretching, which includes the oral and pharyngeal soft tissues. The tongue is surrounded by the mandible and the airway. An enlarged tongue inside a small mandible might move posteriorly and produce a decreased airway.^4^ Additionally, patients with micrognathia are likely to experience airway obstruction by the tongue.^1^

Mandibular size and position are one of the factors that bring about a reduction in tongue space and indirectly will influence the pharyngeal space. Lateral cephalometric studies have been used in an attempt to analyze and identify mandibular morphologic parameters that might be characteristic in adult patients with breathing disorders.^2^ The inter-relationship of mandible, tongue and airway has been studied in Pierre Robin sequence.^4^ But not many studies are found in literature pertaining to the mandibular dimension and tongue in children, which when evaluated, would be correlated with the reduction in pharyngeal airway space. The size ratio norms for tongue and mandible (T/M ratio) are yet to be reported in children suffering from OSA with respect to body mass index (BMI). 

Craniofacial soft tissues can be evaluated by various methods. Imaging plays a role in anatomic assessment of airway and its adjacent structure. It can help in understanding and identifying patients who may be at risk to airway obstructive disorders. But the 2D data may not always totally account for the exact dimensional accuracy. MR imaging offers 3D soft tissue visualization of upper and lower pharyngeal structures along with adjacent structure, like jaws and tongue, hence providing an opportunity to evaluate the functional relationship between them.^5^

Thus, the present study attempted to investigate tongue/mandible ratio in children, using volumetric MRI in an attempt for early diagnosis and to aid in treatment planning. The investigation was carried out with the objectives of evaluating tongue volume/mandible volume ratio (TV/MV ratio) in children with OSA and without OSA (control) using magnetic resonance volumetric imaging, and of evaluating whether a direct correlation exists between TV/MV ratio and BMI in children.

## MATERIAL AND METHODS

The study protocol was approved by Ethical Committee of the Datta Meghe Institute of Medical Sciences University. The volumetric evaluation was carried out retrospectively on available DICOM MR images (using a 1.5 Tesla magnetic resonance imaging scanner, Brivo^TM^ MR355 1.5T; General Electric, Waukesha, WI, USA) for children in the age group of 10-14 years. For the experimental group (OSA), MRI image records for patients diagnosed with OSA (positive polysomnograph) were obtained from the records of the Sleep Apnea, Pulmonary Medicine and Interventional Radiology departments. The age, gender, height and weight of the subjects were retrieved from patient database and registered. A total of 12 scans fulfilling the age criteria and with sufficient diagnostic clarity were included in the study. In the control group, available MRI images of healthy subjects without obstructive sleep apnea were included. The scans were retrieved from patient database at interventional radiology department records and only those who were admitted to any other departments except pulmonary medicine were included as healthy subjects scan. Body mass index (BMI) for both groups was calculated from the height and the weight as per record according to the formula: 

» weight in kilograms/(height in meters)^2^

The image processing and anatomic measurements from MR images was made using DICOM image processing software (OsiriX v. 5.6, Biomedical Visualizers, Geneva, Switzerland). Soft tissue and bony structure segmentation was performed by manual tracing. All the values were rounded off as per decimals, for easy computations. The tongue and mandibular volumes were directly computed using OsiriX software. The software allows the user to designate the Region of Interest (ROI) in each and individual slice. These individual ROIs were then merged to evaluate and compute the volume of the desired anatomical unit. The OsiriX software allows the user to “sculpt out” the desired volume from the rest of the structure.

**Figure 1:**
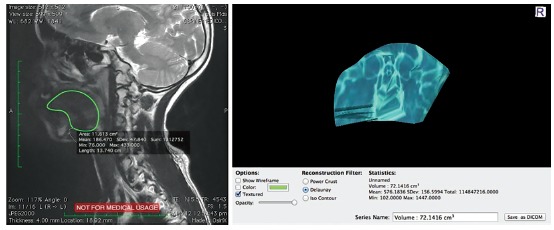
Tongue volume (TV) computation using DICOM image with Osirix.

The volume of the tongue in cm^3^ was calculated from the MRI tracings. The tongue was defined as all of its intrinsic muscles plus the entire genioglossus and hyoglossus muscles (Fig 1).

**Figure 2:**
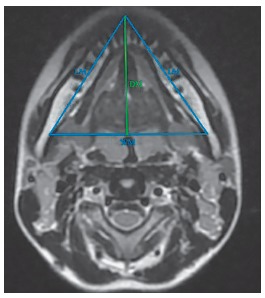
Mandibular volume (MV) computation using DICOM image with Osirix

The mandibular measurements of length, width and depth in centimeters were assessed to determine the dimensions of the lower portion of the mandible. The coordinates for the most anterior-inferior point of the mandible (gnathion) and the most posterior-inferior points of the mandible (left and right gonion) were identified on the corresponding frontal/coronal MRI slices. Mandibular body length (LM) was measured as the average distance from the gnathion to the left and right gonion, and mandibular width (WM) was measured as the distance between the left and right gonion.^5^ Mandibular depth was derived using the measurements as per Pythagoras’ theorem. A triangular representation of the mandible is shown in Figure 2. 

Mandibular depth (DM) was estimated using Pythagoras’ theorem as: DM=√(LM^2^ - {WM/2}^2^).

Thus, the mandibular volume (MV), expressed in cm^3^, was obtained as: MV = 1/3 WM * DM.

The 3D reconstructions were produced using the viewer, and Curved MPR (Curved Multi-Planar Reformation) rendered image from a 3D dataset was obtained. The technique was implemented in this study using OsiriX software by fusing three-dimensional MRI images of the tongue (Fig 1).

The tongue/mandible ratio was generated using the above values, i.e. TV/MV and expressed as a percentage for both the groups.

## RESULTS

Statistical analyses were performed using SPSS version 17.0 for Mac (SPSS Inc; Chicago, Illinois, USA) software. All descriptive statistics are presented as mean ± SD. Descriptive statistics were calculated for age, BMI, tongue volume/mandible volume ratio in experimental and control groups. The obtained data was checked by normalcy curve and it was found to be in the normal range. 

To know the difference between the mean of two groups, unpaired *t*-test was used. Correlation between BMI and TV/MV ratio was analyzed using the Pearson correlation coefficient test. A *p*-value of less than 0.05 was considered to indicate statistical significance. The mean BMI (20.166667 ± 1.078837) and mean TV (82.25 ± 3.319764) were found to be higher in experimental group, as compared to control group (Table 1). The difference between experimental and control groups with respect to TV/MV ratio was found to be highly significant at 0.05 using unpaired *t*-test (*p*= 0.0005, Table 2). There was no significant correlation between BMI and TV/MV ratio in experimental group (*p*= 0.451) as well as control group (*p*= 0.094, Tables 3 and 4).

**Table 1:** Descriptive statistics for body mass index (BMI), tongue volume (TV), and mandibular volume (MV) in experimental and control groups.

Scan number	BMI (kg/m^2^)		TV (cm^3^)		MV (cm^3^)	
	Experimental	Control	Experimental	Control	Experimental	Control
1	19.6	18.6	82	78	1.157	1.216
2	18.4	16.9	81	74	1.280	1.258
3	20.6	18.9	88	80	1.382	1.428
4	20.4	18.8	86	77	1.157	1.178
5	19.5	18.8	81	78	1.088	1.26
6	19.4	17.0	79	76	1.130	1.178
7	19.3	17.6	82	73	1.110	1.224
8	21.9	18.2	85	78	1.080	1.08
9	19.5	18.3	79	81	1.144	1.224
10	20.0	18.4	76	78	1.054	1.13
11	22.1	17.4	82	74	1.140	1.12
12	21.3	17.6	86	79	1.198	1.28
Mean SD	20.166667 ± 1.078837	18.041667 ± 0.683689	82.25 ± 3.319764	77.166667 ± 2.374634	1.16 ± 0.087570	1.214667 ± 0.087007

**Table 2:** Descriptive statistics for TV/MV ratio in experimental and control groups.

TV/MV ratio	n	Mean ± SD	Std. error mean	Mean difference	95% Confidence interval of the difference		p-value
					Lower	Upper	
Experimental group	12	71.1647 ± 4.38150	1.26483	71.16468	68.3808	73.9485	0.0005*
Control group	12	63.8027 ± 4.49868	1.29866	63.80271	60.9444	66.6610	

*Significant for *p* < 0.05.

**Table 3:** Pearson correlations between BMI and TV/MV ratio in experimental group.

		TV/MV ratio experimental group	BMI experimental group
TV/MV ratio experimental group	Pearson correlation	1	0.451
	Sig. (2-tailed)		0.141
	n	12	12
BMI experimental group	Pearson correlation	0.451	1
	Sig. (2-tailed)	0.141	
	n	12	12

**Table 4:** Pearson correlations between BMI and TV/MV ratio in control group.

		TV/MV ratio control group	BMI control group
TV/MV ratio control group	Pearson correlation	1	0.094
	Sig. (2-tailed)		0.771
	n	12	12
BMI control group	Pearson correlation	0.094	1
	Sig. (2-tailed)	0.771	
	n	12	12

## DISCUSSION

According to the Functional Matrix Theory of Moss,^6^ there is close interrelationship in the functions of swallowing and breathing. Humans are predominantly nasal breathers. In case of breathing or airway obstruction, the body adopts one or all three neuromuscular responses i.e. an altered mandibular posture, an altered tongue posture and an extended head posture. In children with obstructive breathing diseases, the most common etiologic factors may be related with hypertrophic tonsils, alterations in craniofacial growth, retrognathia, macroglossia, obesity and neurological lesions.^7^

With respect to craniofacial parameters playing role in OSA: since the mandible and the airway surround the tongue, and the tongue occupies the space between the maxillary and mandibular dental arches when the mandible is in its physiologic rest position; the presence, size and activity of this large muscular organ has a definite moulding and shaping influence on the form and size of the palate and the dental arches. An enlarged tongue inside a small mandible might move posteriorly and produce a decreased airway. Obesity has always been thought to be a confounding factor in OSA. The relation between BMI (as obesity is a common etiologic factor for OSA) and the ratio of tongue volume and mandible volume (TV/MV ratio) has not yet been reported in children. In this study, the correlation of TV/MV ratio and BMI was investigated with a 3D reconstructed model from MRI data.

The subjects in the age range of 10-14 years were selected to observe soft tissue and hard tissue differences. Nearly 90% of the maxillary and mandibular growth is completed by 12 years of age, so most of the formation or deformation occurs by that age. The dimensions of the pharynx continue to grow rapidly until 12 years of age and then slow until adulthood.^8^ In the present study, based on these data, the age group of growing children was selected to evaluate effect of tongue and mandible on breathing in OSA.

Tongue volume is an important parameter analyzed in the present study. This has been of considerable interest as it plays a central regulatory role in the craniofacial complex development. Song and Pae^9^ suggested that enlarged tonsils increase the upper airway resistance, which might facilitate the activity of oropharyngeal muscles (genioglossus, mylohyoid, etc.) through mechanoreceptors in the upper airway. This action would bring the tongue forward and downward, opening the mouth for better respiration. And it could also result in a downward and backward rotation of the mandible. In the present study, largest mean of tongue volume was found to be in OSA group. With these findings we can presume that increased tongue volume can probably be a craniofacial anatomic risk factor for development of breathing disorders and OSA. But this does not rule out that patients with smaller tongue volumes relative to oral cavity size may not have other (e.g. non-anatomic) factors predisposing to breathing disorders and apnea. This possibility demands further investigation. 

Our findings also confirmed that due to an increased tongue volume, more tongue mass would occupy the upper airway space, and this in turn could make the upper airway more prone to obstruction. These findings were corroborated by the findings of Iida-Kondo et al,^1^ who reported that patients with sleep-disordered breathing tended to have larger tongues, compared to patients without sleep-disordered breathing.

Our study also evaluated the mandibular dimension and volume. The observations by Solow et al^3^^,^^10^ supported that the airway adequacy was related to the size and position of the mandibular variable rather than maxillary variables. However, as per our findings, it was not found to be significantly different between the two groups. Our findings were contradictory, which could be attributed to the ethnic differences and unique anthropological features in Indian population.

Adding another dimension to the present investigation, we derived the mandibular area measurements according to the Pythagoras’ theorem. In an attempt to find out the correlation between BMI and TV/MV ratio, we carried out a correlation analysis. However, no significant correlation was found. The primary risk factors for OSA are either obesity or having an abnormal upper-airway anatomy. In previous studies, the soft and hard tissue structures were analyzed with cephalometric images. Yu et al^11^ reported that obese OSA patients had a longer tongue than did simple snorers and non-obese OSA patients. However, these early reports were limited to the analyses of data obtained from the sagittal view. Recent studies have demonstrated that 3-dimensional (3D) MRI and Cone-Beam Computed Tomography techniques performed while the patient is awake are suitable for evaluation of upper airway volume in OSA patients. Schwab et al^12^ analyzed the upper airway soft tissue structures 3-dimensionally with an advanced analysis technique via MR imaging. They concluded that the volume of the tongue and lateral pharyngeal walls were shown to independently increase the risk of sleep apnea. On the other hand, Okubo et al^13^ carried out a similar study and reported that the tongue volume was not significantly different between OSA and controls, and the tongue volume did not correlate with BMI. The results of the present study are corroborated by Okubo et al.^13^ Recent evidence suggests that the mechanisms underlying apnea are highly variable, with some patients having primarily an anatomic problem. It has been shown that in addition to individual anatomic factors, relationships between soft-tissue and bony enclosure size are altered, a concept termed anatomic balance. 

In 2006, Iida-Kondo et al^1^ compared the tongue volume/oral cavity volume (TV/OCV) ratio between 20 male patients with OSA and 20 normal male adults. They described that BMI was significantly correlated with tongue volume in the OSA patient group, which is not consistent with our results. In the present study, the BMI was found to be consistently lower, in average 10% less in the control group. An increased tongue volume was found in OSA group; however, the mandible volume was found to be similar in both groups.

We thus presume that in OSA patients, the mandible is less able to properly accommodate the increased tongue volume. As a result, the enlarged tongue moves posteriorly, decreasing the airway volume. As tongue volume increases, the airway volume decreases and thus is likely to be involved in the development of OSA.

Based on the present results, we assume that craniofacial morphology is indeed an important factor that could contribute to collapsing upper airways during sleep. Craniofacial morphologic abnormalities fall into two principal categories: skeletal anomalies, such as a small jaw; and soft tissue anomalies, such as enlargement of the soft palate or tongue area. Skeletal morphology is determined by genetic and developmental factors, whereas the morphology of soft tissue such as the tongue is related to body mass index (BMI). Thus, craniofacial morphology as a risk assessment of OSA should be determined based on a balance between the jaw as a container and soft tissue as its content, as investigated in the present study through TV/MV ratio. 

## LIMITATIONS

Prospective studies, with a larger sample size and including subjects from different ethnic populations would give a better outlook towards the delicate craniofacial anatomic balance disruption in OSA.

## CONCLUSION 

We conclude that TV/MV ratio may be an appropriate variable to screen risk factors for OSA (within the scope of the present paper), representing the balance between skeletal morphology and soft tissue morphology in craniofacial complex. 

The acquired knowledge on tongue/mandible volumetric ratio can be utilized clinically as a diagnostic aid in determining the anatomic risk factors in craniofacial region for development of OSA at an early age. The obtained values could be a guiding factor for further large-scale studies to correlate severity of OSA with respect to BMI, age and other biological parameters. 
